# Aquaporin3 Is Required for FGF-2-Induced Migration of Human Breast Cancers

**DOI:** 10.1371/journal.pone.0056735

**Published:** 2013-02-28

**Authors:** Xu-Chen Cao, Wei-Ran Zhang, Wen-Feng Cao, Bo-Wen Liu, Fei Zhang, Hong-Meng Zhao, Ran Meng, Lin Zhang, Rui-Fang Niu, Xi-Shan Hao, Bin Zhang

**Affiliations:** 1 National Key Laboratory of Breast Cancer Prevention and Treatment, Cancer Institute and Hospital, Tianjin, China; 2 Department of Breast Cancer Surgery, Cancer Institute and Hospital, Tianjin, China; 3 Department of Pathology, Cancer Institute and Hospital, Tianjin, China; 4 Tianjin Key Laboratory of Cancer Prevention and Treatment, Tianjin Medical University, Tianjin, China; INRS, Canada

## Abstract

**Purpose:**

The aquaporin (AQP) family consists of a number of small integral membrane proteins that transport water and glycerol. AQPs are critical for trans-epithelial fluid transport. Recent reports demonstrated that AQPs, particularly AQP1 and AQP5, are expressed in high grade tumor cells of a variety of tissue origins, and that AQPs are involved in cell migration and metastasis. Based on this background, we examined whether AQP3, another important member of the AQP family, could facilitate cell migration in human breast cancers.

**Methods:**

Potential role of AQP3 was examined using two representative breast cancer cell lines (MDA-MB-231 and Bcap-37). Briefly, AQP3 expression was inhibited with a lentivirus construct that stably expressed shRNA against the AQP3 mRNA. AQP3 expression inhibition was verified with Western blot. Cell migration was examined using a wound scratch assay in the presence of fibroblast growth factor-2 (FGF-2). In additional experiments, AQP3 was inhibited by CuSO_4_. Fibroblast growth factor receptor (FGFR) kinase inhibitor PD173074, PI3K inhibitor LY294002, and MEK1/2 inhibitor PD98059 were used to dissect the molecular mechanism of FGF-2 induced AQP3 expression.

**Results:**

FGF-2 treatment increased AQP3 expression and induced cell migration in a dose dependent manner. Silencing AQP3 expression by a lentiviral shRNA inhibited FGF-2 induced cell migration. CuSO_4_, a water transport inhibitor selective for AQP3, also suppressed FGF-2-induced cell migration. The FGFR kinase inhibitor PD173074, significantly inhibited FGF-2-induced AQP3 expression and cell migration. The PI3K inhibitor LY294002 and MEK1/2 inhibitor PD98059 inhibited, but not fully blocked, FGF-2-induced AQP3 expression and cell migration.

**Conclusions:**

AQP3 is required for FGF-2-induced cell migration in cultured human breast cancer cells. Our findings also suggest the importance of FGFR-PI3K and FGFR-ERK signaling in FGF-2-induced AQP3 expression. In summary, our findings suggest a novel function of AQP3 in cell migration and metastasis of breast cancers.

## Introduction

Breast cancer is the most prevalent malignancy and the second leading cause of cancer death in women. Significant progress has been made in tumor detection and treatment. However, metastasis remains a significant cause of morbidity and mortality of this disease [1].

Growth factors control tumor cell invasion and migration, and therefore ultimately metastasis. FGF-2 is one of those growth factors that are associated with cancer development, and has been demonstrated to be an essential regulator of epithelial cell proliferation, migration and angiogenesis [2]. The biological activities of FGF-2 are meditated by a dual receptor system consisting of high-affinity tyrosine kinase receptor and low affinity binding sites corresponding to heparan sulfate proteoglycans (HSPN) [3]. The formation of active FGF-FGFR complex is the prerequisite for effective intracellular signaling.

The aquaporin (AQP) family consists of a number of membrane proteins that transport water and glycerol [4]. Currently, there are at least 13 identified members in mammalian cells. AQPs are critical in trans-epithelial fluid transport, and therefore urine concentration and exocrine gland secretion [5]–[7]. AQPs are involved in other, and sometimes unexpected functions, for instance, neural signal transduction and fat metabolism [8], [9].

Increasing evidences from both *in vitro* and *in vivo* experiments suggested that AQPs could facilitate tumor cell migration. AQP5 could promote cell migration and proliferation in SPC-A1 lung cancer cells [10]. Over-expression of AQP1 increases the metastatic potential of breast cancer cells [11]. AQPs-facilitated cell migration has also been found in colon, ovary, and brain cancer cells [12]–[14]. A potential consequence of AQP-mediated cell migration is enhanced metastatic potential through accelerating cell migration across microvessels and into normal tissues [11]. In clinical studies [15], [16], AQPs expression has been found to be correlated with tumor metastasis and prognosis.

A recent study of human breast cancer showed that, among the 13 AQP members, only AQP1, AQP3, and AQP5 expression is elevated in breast cancer tissues relative to normal tissues [17]. Previous studies suggested that AQP1 could facilitate cell migration in 4T1 breast cancer cells [11], and that AQP5 is required for proliferation and migration in MCF-7 breast cancer cells [18]. However, few reports focused on the potential role of AQP3 in breast cancer. The current study tested the hypothesis that AQP3 could facilitate FGF-2-induced cell migration. We also speculate that FGF-2 upregulates AQP3 expression via FGFR-PI3K and FGFR-ERK signalings.

## Materials and Methods

### Cell Culture

Human breast cancer cell lines MDA-MB-231 (Rockville, MD, USA) and Bcap-37 (Tianjin Medical University Cancer Institute and Hospital, Tianjin, China) were cultured in RPMI-1640 medium (Hyclone, Logan, Utah, USA) with 10% fetal bovine serum (Hyclone), supplemented with 1% penicillin/streptomycin, in a 5% CO_2_ and humidified atmosphere at 37°C.

### Antibodies and Reagents

The rabbit anti-AQP3 and mouse anti-β-actin were obtained from Santa Cruz Biotechnology (Santa Cruz, CA, USA). Rabbit anti-phospho-AKT, rabbit anti-AKT, rabbit anti-phospho-ERK1/2, rabbit anti-ERK1/2, goat anti-rabbit IgG-HRP and goat anti-mouse IgG-HRP were from Cell Signaling Technology (Beverly, MA, USA). FGF-2 and CuSO_4_ were from Sigma (St. Louis, MO,USA). PD173074, LY294002, and PD98059 were from Selleck Chemicals (Houston, TX, USA).

### Wound Scratch Assay

The 6-well plate was precoated with polylysine (30 µg/ml), followed by BSA blocking. Cells (1×10^6^) were seeded in a 6-well plate and cultured as monolayer to confluence overnight prior to serum starvation for 24 h. The monolayer was then scratched with a pipette tip, and washed twice with PBS to remove floating cells. After the line scratch, cells were incubated for various time periods (from 6 h to 24 h), in the presence of mitomycin C (10 µg/ml) to prevent cell proliferation. Cell migration was expressed as the percentage of the gap relative to the total area of the cell-free region immediately after the scratch using an ImageJ software (National Institutes of Health, Bethesda, MD, USA). For each plate, 5 randomly selected images were acquired. All experiments were carried out in triplicate.

### Western Blot Analysis

After PBS washing, cells were harvested by scraping into 150 µl RIPA buffer (containing 50 mM Tris-HCl, pH 7.4, 150 mM NaCl, 1% NP-40, 1 mM EDTA, 0.25% sodium deoxycholate), 1 mM NaF, 10 µM Na_3_VO_4_, 1 mM PMSF (phenyl -methylsulfonylfluoride), and protease inhibitor cocktail (10 µg/ml leupeptin, 10 µg/ml aprotinin, and 1 µM pepstatin). After incubation at 4°C for 30 min, the lysates was centrifuged at 15000 g for 10 min at 4°C. Protein concentration was determined using a BCA assay (Pierce, Rorkford, IL, USA). Samples (30 mg protein) were denatured in 5×SDS/PAGE sample buffer for 5 min at 95°C, and subjected to 10% SDS/PAGE. The separated proteins were transferred on to a PVDF membrane (Millipore, Bedford, MA, USA) for 2 h at 4°C. Non-specific binding was blocked by 5% dry milk in TBST (20 mM Tris/HCl, 137 mM NaCl and 0.05% Tween 20, pH 7.4) overnight at 4°C. After blocking, membranes were incubated with one of the primary antibodies against AQP3 (1∶1000), AKT (1∶1000), p-AKT (1∶1000), ERK1/2 (1∶1000), p-ERK1/2 (1∶1000) or β-actin (1∶10000) in diluted buffer (3% BSA in TBS) over night at 4°C. The blots were then incubated with an appropriate secondary antibody (HRP-conjugated anti-goat, anti-rabbit or anti-mouse secondary antibody) at appropriate dilution at room temperature for 1 h, and quantified using an ECL kit (Pierce, Rorkford, IL, USA). Band density was quantified using an ImageJ software (National Institutes of Health). All data were normalized to β-actin for the final analyses.

### Lentivirus-mediated RNA Interference Experiments

MISSION lentiviral transduction particles encoding shRNAs targeting AQP3 or control were purchased from Sigma. Briefly, cells were cultured in complete medium without antibiotics for 2 days, and seeded in a 6-well plate at a density of 1×10^5^ cells per well. Upon 30%–50% confluence, cells were infected with lentiviral -scrambled-shRNA or lentiviral-AQP3-shRNA at a multiplicity of infection (MOI) of 20. Stable cell lines were selected with 2 µg/ml puromycin (Sigma-Aldrich, St. Louis, MO, USA) for one week to eliminate uninfected cells. Then the medium was replaced, and after 2 days, cells were harvested for Western blot analysis.

### Statistical Analysis

Data are presented as mean ± SEM, and analyzed by one-way analysis of variance (ANOVA). All experiments were carried out for at least three times independently. Statistical significance was set at *P*-values <0.05.

## Results

### FGF-2 Induces AQP3 Expression in Cultured Human Breast Cancer Cell Lines

**Figure 1:**
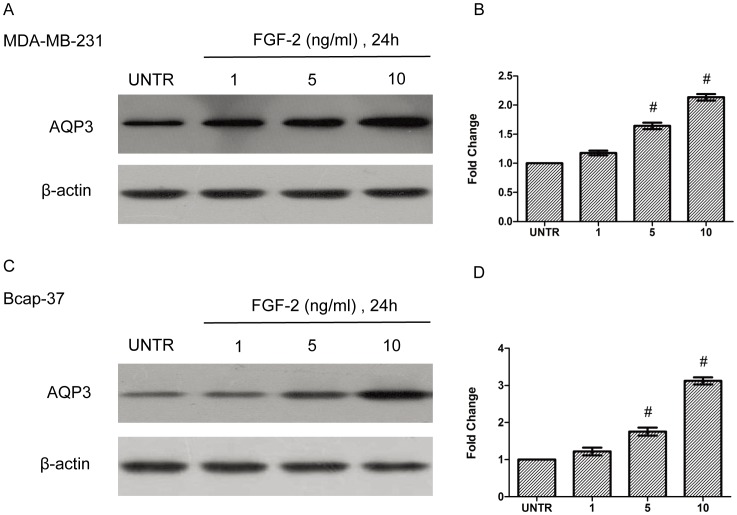
FGF-2 induces AQP3 expression in human breast cancer cell lines. MDA-MB-231 and Bcap-37 cells were treated with FGF-2 (0, 1, 5, or 10 ng/ml) for 24 h. AQP3 expression was analyzed by Western blot and normalized to β-actin. The data represented mean ± SEM for triplicate experiments. ^#^ *P*<0.05 versus untreated condition (UNTR).

First, we tested whether FGF-2 induces AQP3 up-regulation in human breast cancers. MDA-MB-231 and Bcap-37 were treated with FGF-2 at concentrations of 0, 1, 5, 10 ng/ml. The cell lysates were then analyzed for AQP3 by Western Blot. FGF-2 increased AQP3 protein in a dose-dependent manner (Figs. 1A and 1C). FGF-2-induced AQP3 expression was increased by 1.1-, 1.6- and 2.1-fold in MDA-MB-231 and 1.2-, 1.8- and 3.1-fold in Bcap-37 cells, respectively (Figs. 1B and 1D).

### FGF-2 Induces Cell Migration in Cultured Human Breast Cancer Cells

**Figure 2:**
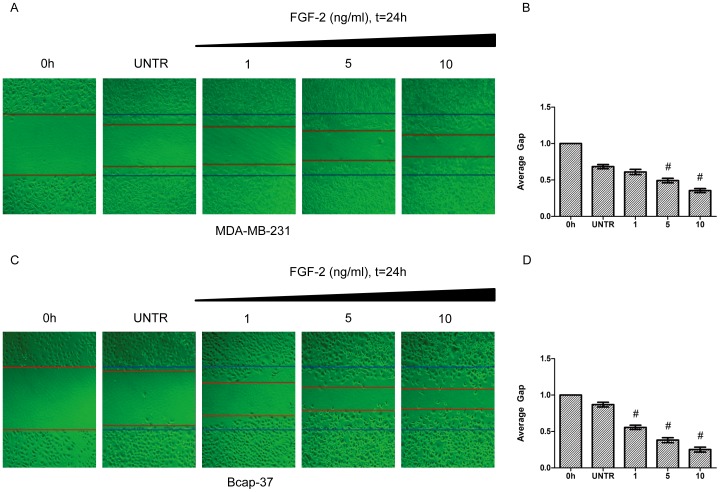
FGF-2 induces cell migration in human breast cancer cell lines. MDA-MB-231 and Bcap-37 were treated with FGF-2 at concentrations of 0, 1, 5, 10 ng/ml. Cell migration was detected by wound scratch assay and photographed at 24 h, as shown in A and C. Cell migration were represented as average gap in B and D. The results represent mean ± SEM for triplicate experiments. For cell migration experiment, at least 50 cell migration distances were counted for one experiment. ^#^ *P*<0.05 versus untreated groups (UNTR).

We next investigated whether FGF-2 induces cell migration in the two representative breast cancer cell lines. MDA-MB-231 and Bcap-37 were treated with FGF-2 at concentrations of 0, 1, 5 and 10 ng/ml. As shown in Figure 2A and 2C, the starved untreated cells exhibited only a limited wound closure activity. In contrast, the FGF-2-treated cells showed acceleration of wound closure that could be observed after treatment with various concentrations of FGF-2. The average gap was decreased by 0.61-, 0.49-, and 0.35-fold in MDA-MB-231 and 0.56-, 0.38-, and 0.25-fold in Bcap-37, respectively (Figs. 2B and 2D).

### Lentivirus-mediated shRNA Inhibits AQP3 Expression in Human Breast Cancer Cell Lines

**Figure 3:**
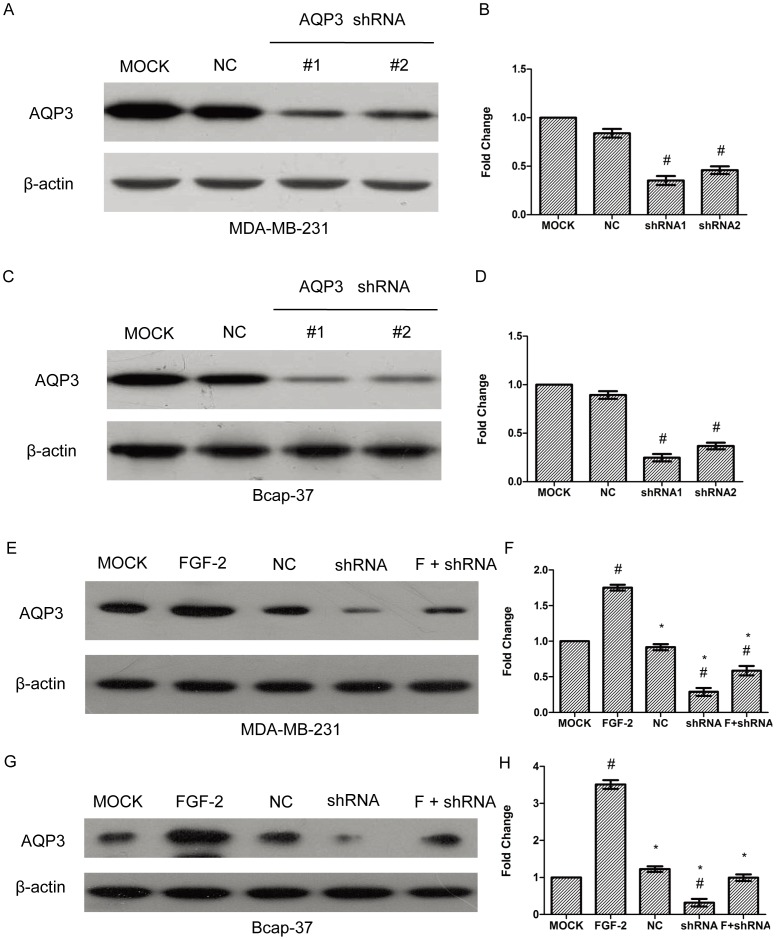
Lentivirus-mediated shRNA inhibits AQP3 expression. Two shRNA-expressing lentivirus vector were transfected into MDA-MB-231 and Bcap-37 cells. After the selection of cells that could stably express shRNA, AQP3 expression was analyzed by Western blot and normalized to β-actin (A to D). A scramble sequence was used as the negative control (NC). In figure E to H, MDA-MB-231 and Bcap-37 cells were stably transfected with lentiviral shRNA1 against AQP3, followed by treatment with or without FGF-2 (10 ng/ml) for 24 h. A scramble sequence was used as the negative control (NC). AQP3 expression was analyzed by Western blot, and normalized to β-actin. The data represented mean ± SEM for triplicate experiments. ^#^ *P*<0.05 versus MOCK.*P<0.05 versus FGF-2 alone.

To verify the specificity of the intervention, cells were transfected with two different shRNA targeting AQP3 before further analysis. Stable transfection of MDA-MB-231 and Bcap-37 cells with two constructs inhibited AQP3 expression by 65% and 75% (shRNA1) and 54% and 63% (shRNA2) in MDA-MB-231 and Bcap37 cells, respectively (Figs. 3A to 3D). Based on this finding, shRNA1 was used in the following experiments.

### Gene Silence of AQP3 and CuSO_4_ Inhibit FGF-2-induced Cell Migration in Human Breast Cancer Cell Lines

**Figure 4:**
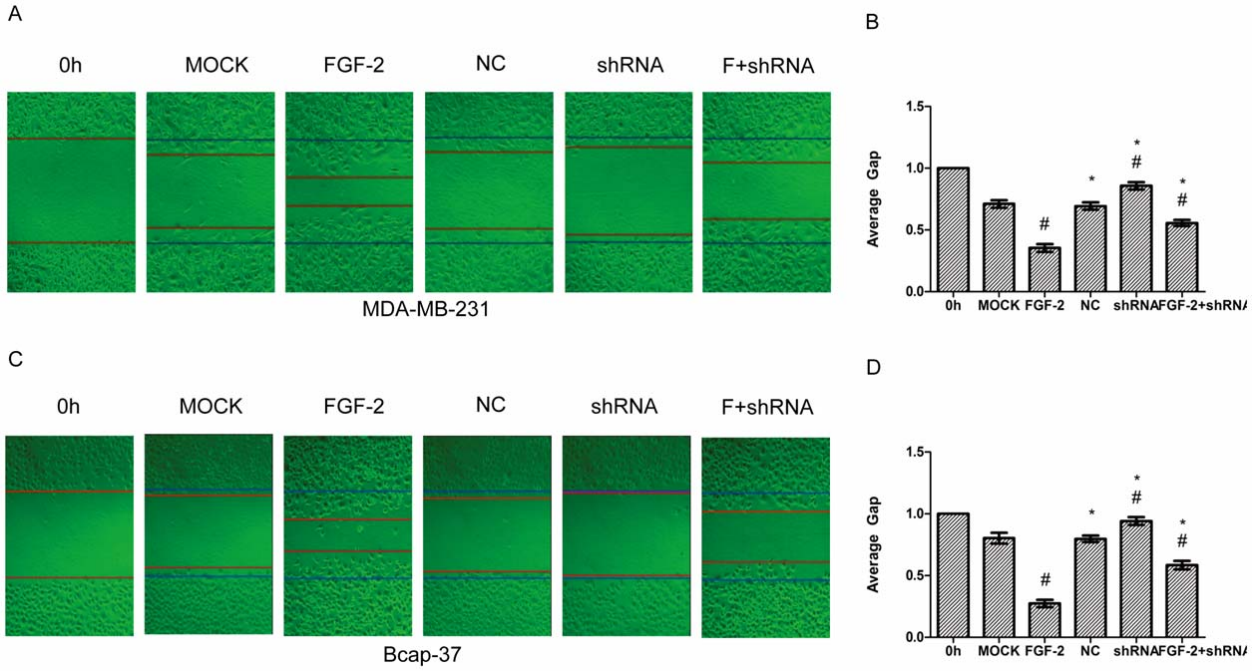
AQP3 silencing reduces FGF-2-induced cell migration. MDA-MB-231 and Bcap-37 cells were stably transfected with lentiviral shRNA against AQP3, followed by treatment with or without FGF-2 (10 ng/ml) for 24 h. A scramble sequence was used as the negative control (NC). Cell migration was examined using wound scratch assay, photographed at 24 h, and represented as average gap (B and D). The results represent means ± SEM for triplicate experiments. For the cell migration experiment, at least 50 cell migration distances were counted for each experiment. ^#^ *P*<0.05 versus MOCK. *P<0.05 versus FGF-2 alone.

**Figure 5:**
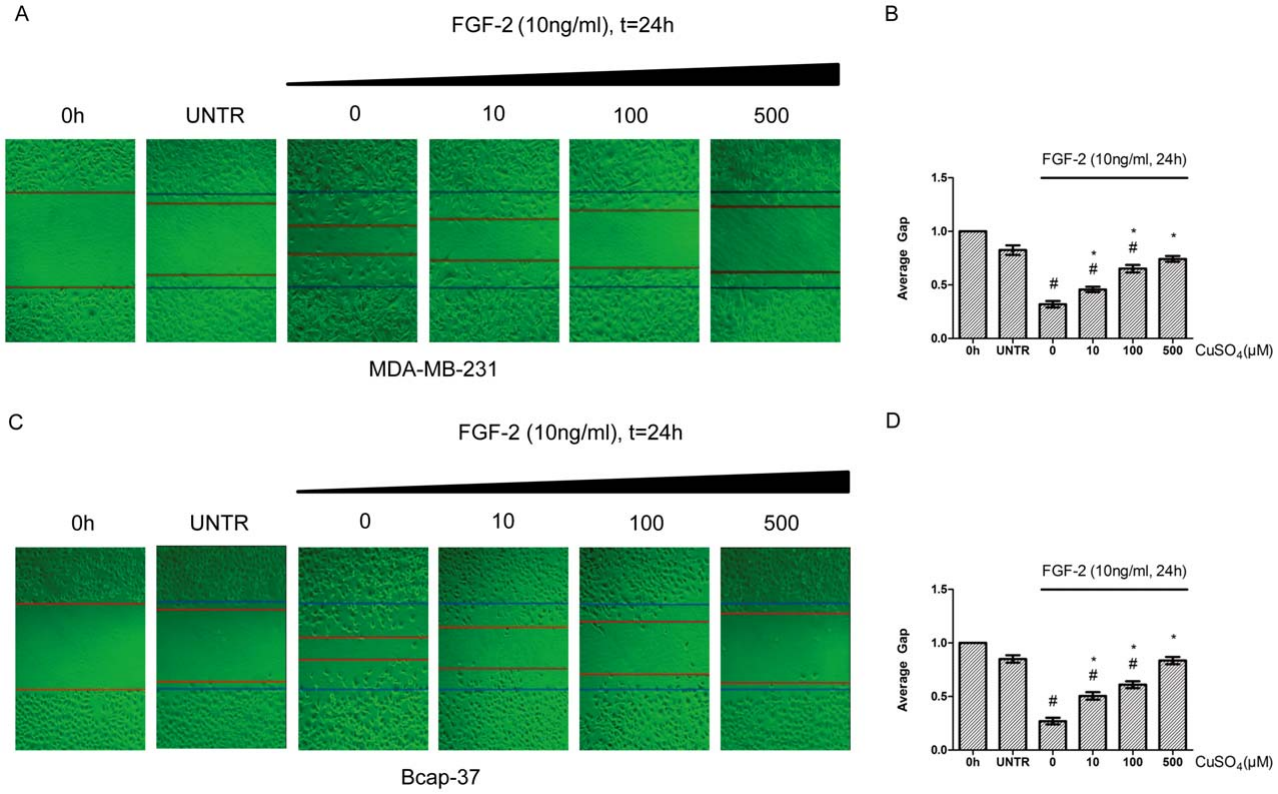
CuSO_4_ inhibits FGF-2-induced cell migration. MDA-MB-231 and Bcap-37 cells were treated with FGF-2 (10 ng/ml) and CuSO_4_ (0, 10, 100, or 500 µM). Cell migration was examined using wound scratch assay, photographed at 24 h, and quantified as average gap (B and D). The results represented mean ± SEM for triplicate experiments. For cell migration experiment, at least 50 cell migration distance were counted for each experiment. ^#^ *P*<0.05 versus untreated control (UNTR). *P<0.05 versus FGF-2 alone.

To investigate whether AQP3 is involved in FGF-2 induced cell migration, RNAi experiment was performed. As expected, silencing AQP3 gene with a lentiviral shRNA (shRNA1) led to a prominent decrease of AQP3 basal expression, and inhibited FGF-2-induced AQP3 expression by 67% in MDA-MB-231 and 72% in Bcap-37 cells, respectively (Figs. 3E to 3H). The scramble control shRNA had no effect. Silence of the AQP3 gene also reduced FGF-2-induced cell migration (Figs. 4A and 4C). The shRNA treatment increased the average gap from 0.35-fold (FGF-2alone) to 0.56-fold (FGF-2 plus shRNA) in MDA-MB-231, and from 0.27-fold to 0.58-fold in Bcap-37 cells, respectively (Figs. 4B and 4D). Treatment with the scramble control shRNA had no effect. To further identify the role of AQP3 in FGF-2-induced cell migration, CuSO_4_, a water transport inhibitor of AQP3, was included. CuSO_4_ treatment at 10–500 µM decreased migration of MDA-MB-231 and Bcap-37 cells induced by 10 ng/ml FGF-2 (Figs. 5A and 5C). The inhibitory effect of CuSO_4_ was apparent at a concentration of 10 µM and was most prominent at 500 µM in both breast cancer cell lines (Figs. 5B and 5D).

### FGFR Mediates AQP3 Expression and Cell Migration in Human Breast Cancer Cell Lines

**Figure 6:**
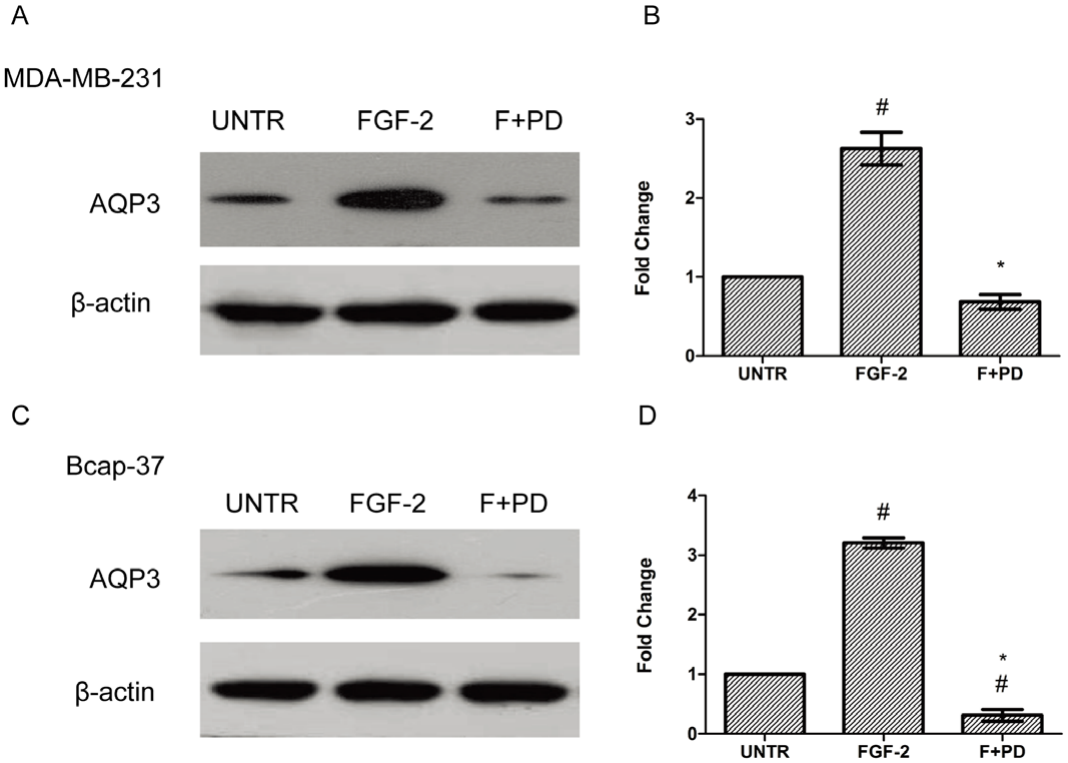
PD173074 significantly inhibits FGF-2-induced AQP3 expression. MDA-MB-231 and Bcap-37 cells were treated with FGF-2 (10 ng/ml) with or without FGFR kinase inhibitor PD173074 (1 µM) for 24 h. AQP3 expression was analyzed by Western Blot and normalized to β-actin. The results represent means ± SEM for triplicate experiments. ^#^ *P*<0.05 versus untreated control (UNTR). *P<0.05 versus FGF-2 alone.

**Figure 7:**
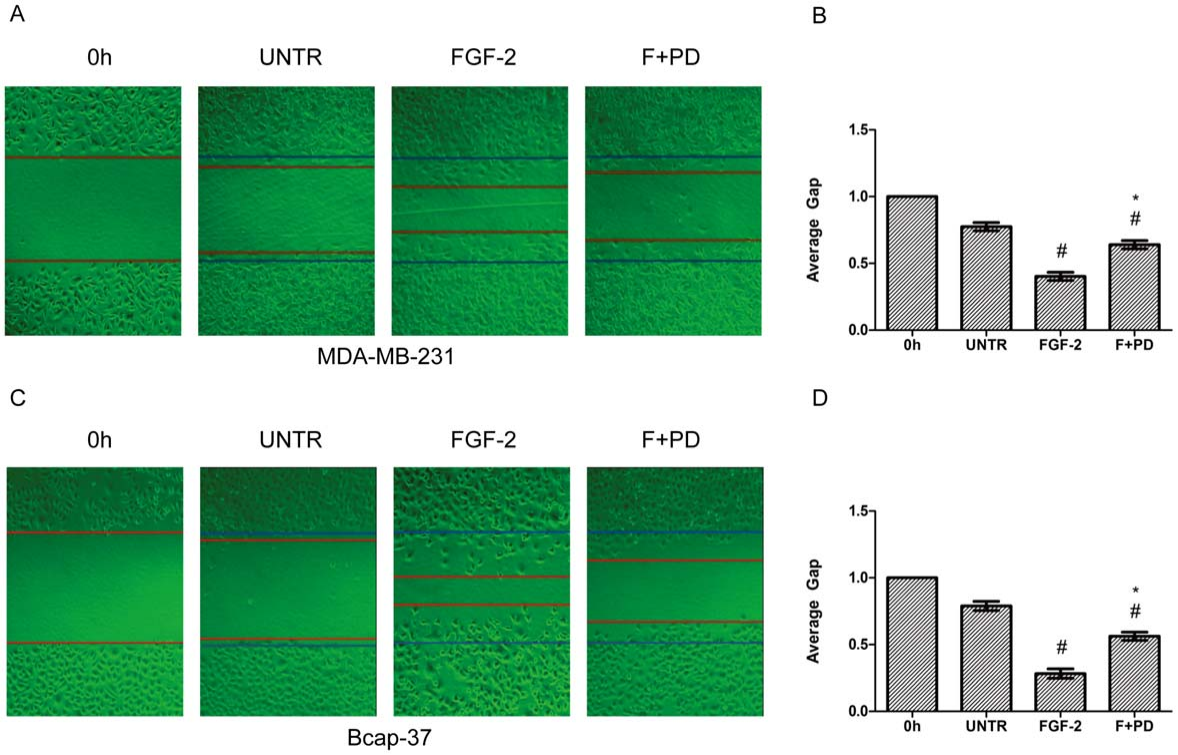
PD173074 significantly reduces FGF-2-induced cell migration. MDA-MB-231 and Bcap-37 cells were treated with FGF-2 (10 ng/ml) with or without FGFR kinase inhibitor PD173074 (1 µM) for 24 h. Cell migration was examined using wound scratch assay, photographed and quantified as average gap (B and D). For cell migration experiment, at least 50 cell migration distance were counted for each experiment. The results represent means ± SEM for triplicate experiments. ^#^ *P*<0.05 versus untreated control (UNTR). *P<0.05 versus FGF-2 alone.

Additional experiments were performed to study the signal pathway of FGF-2- induced AQP3 expression. To better comprehend the role of FGFR in FGF-2-induced AQP3 expression, PD173074, a FGFR kinase inhibitor, was used in the experiment. PD173074 significantly inhibited FGF-2-induced AQP3 expression (Figs. 6A and 6C). PD173074 decreased AQP3 expression by 74% and 90% in MDA-MB-231 and Bcap-37 cells, respectively (Figs. 6B and 6D). FGF-2-induced cell migration was also inhibited by PD173074 in both cell lines (Figs. 7A and 7C). The average gap was increased from 0.40-to 0.64-fold and from 0.28-to 0.57-fold in MDA-MB-231 and Bcap-37 cells, respectively (Figs. 7B and 7D).

### PI3K and ERK Pathways are Involved in FGF-2-induced AQP3 Expression and Cell Migration

**Figure 8:**
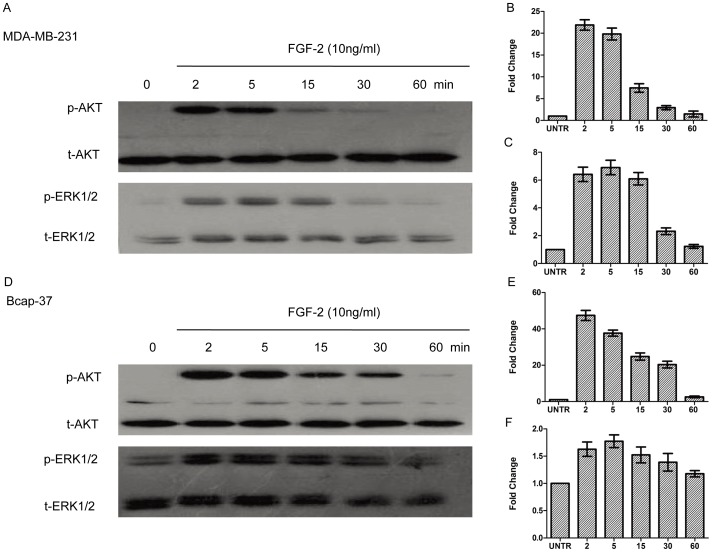
FGF-2 induces AKT and ERK1/2 phosphorylation. For AKT and ERK1/2 phosphorylation, cells were treated with FGF-2 (10 ng/ml) and harvested at (2,5, 15, 30, 60 min). Phospho-AKT (p-AKT) and phospho-ERK1/2 (p-ERK1/2) were analyzed by Western blot and normalized to total AKT(t-AKT) and total-ERK1/2 (t-ERK1/2), respectively. The results represented mean ± SEM for triplicate experiments.

**Figure 9:**
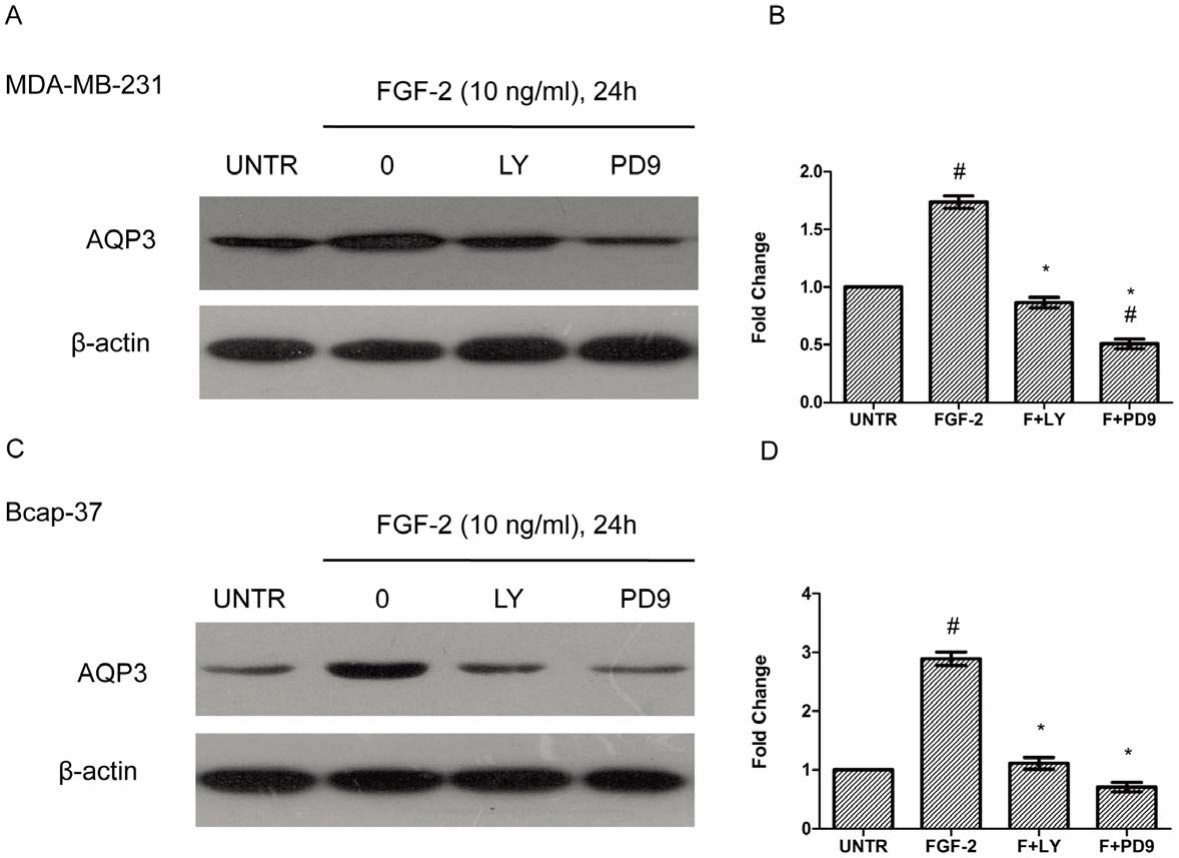
The PI3K and ERK pathways are involved in FGF-2-induced AQP3 expression. MDA-MB-231 and Bcap-37 cells were treated with FGF-2 (10 ng/ml), plus PI3K inhibitor LY294002 (10 µM) and MEK1/2 inhibitor PD98059 (10 µM) for 24 h. AQP3 expression was analyzed by Western blot and normalized to β-actin. The results represented mean ± SEM for triplicate experiments. ^#^ *P*<0.05 versus untreated control (UNTR). *P<0.05 versus FGF-2 alone.

**Figure 10:**
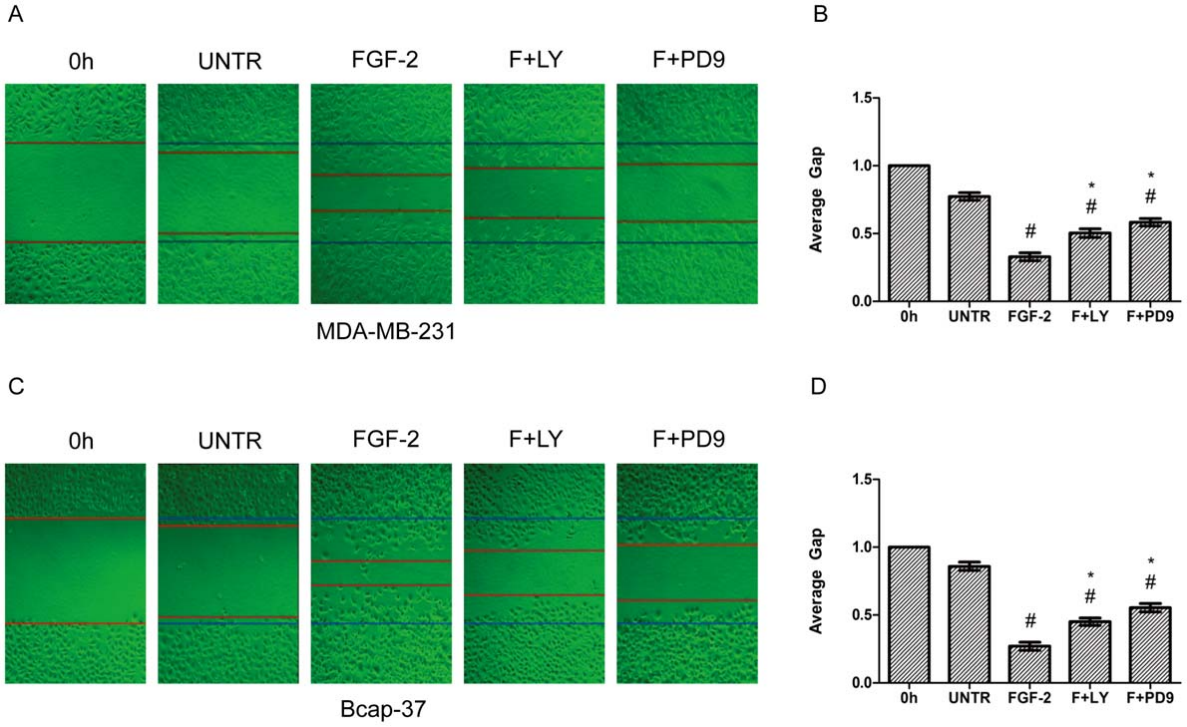
The PI3K and ERK pathways are involved in FGF-2-induced cell migration. MDA-MB-231 and Bcap-37 cells were treated with FGF-2 (10 ng/ml), plus PI3K inhibitor LY294002 (10 µM) and MEK1/2 inhibitor PD98059 (10 µM) for 24 h. Cell migration was examined by wound scratch assay, photographed at 24 h, and quantified as average gap. The results represented mean ± SEM for triplicate experiments. For cell migration experiment, at least 50 cell migration distance were counted for each experiment. ^#^ *P*<0.05 versus untreated control (UNTR). *P<0.05 versus FGF-2 alone.

To further investigate the signal pathway of FGF-2-induced AQP3 expression and cell migration, additional experiments were conducted using kinase inhibitors. As expected, FGF-2 induced AKT and ERK1/2 phosphorylation (Figs. 8A and 8D). ERK1/2 phosphorylation reached a peak at 5 min after FGF-2 treatment; AKT phosphorylation peaked as early as 2 min; the phosphorylation of AKT and ERK1/2 remained sustained for 1 h (Figs. 8B, 8C, 8E and 8F). Western blot analyses showed that pretreatment of PI3K inhibitor LY294002 or MEK1/2 inhibitor PD98059 inhibited, but not fully blocked FGF-2-induced AQP3 expression (Figs. 9A and 9C). In MDA-MB-231 cells, AQP3 expression was decreased from 1.74-(FGF-2alone) to 0.87-fold by LY294002 and 0.51-fold by PD98059. In Bcap-37 cells, AQP3 expression was reduced from 2.89-(FGF-2 alone) to 1.11-fold by LY294002 and 0.71-fold by PD98059. Likewise, FGF-2-induced cell migration was also reduced (Figs. 10A and 10C). In MDA-MB-231 cells, the average gap was increased from 0.32-(FGF-2 alone) to 0.50-fold by LY294002 and0.58-fold by PD98059. In Bcap-37 cells, the gap was increased from 0.27-(FGF-2alone) to 0.45-fold by LY294002 and 0.55-fold by PD98059 (Figs. 10B and 10D).

## Discussion

Previous studies have demonstrated elevated expression of AQPs (AQP1, 3 and 5) in breast cancer tissues relative to adjacent normal tissues [17]. In clinical studies, AQP1 expression is correlated with tumor grade and poor prognosis [19]. AQP5 is associated with cellular differentiation, lymph node invasion, and clinicopathological staging [20]. In addition, AQP1 could facilitate cell migration in 4T1 breast cancer cells [11], AQP5 is required for proliferation and migration in MCF-7 breast cancer cells [18]. Put together, these findings suggested AQPs expression is correlated with cell migration and metastatic potential in human breast cancer. However, little is known about the significance of AQP3 in breast cancer.

Here, we hypothesized that AQP3 in breast cancer could facilitate FGF-2-induced cell migration. To better probe into the mechanisms of this progress, we first investigated whether FGF-2 could induce AQP3 expression in human breast cancer cell lines. The results demonstrated that FGF-2 could up-regulate AQP3 in a dose-dependent manner in both MDA-MB-231 and Bcap-37 cell lines.

Cell migration is a multistep process involving numerous soluble growth factors, cytokines and proteases, as well as extracellular matrix proteins [21], [22]. FGF-2 and its receptors are widely distributed in tumoral tissues [23], [24], and are implicated in epithelial cell proliferation, migration and angiogenesis [2]. FGF-2 signaling that contributes to cell migration has been proven to be important in tumor progression and metastasis [25]–[28]. Our results by using wound scratch assay confirmed that FGF-2 could increase cell migration in both MDA-MB-231 and Bcap-37 breast cancer cells.

It is notable that the FGF-2-induced AQP3 up-regulation coincided with the FGF-2-induced migration in the two representative breast cancer cell lines. In the next set of experiment, we demonstrated that silencing AQP3 gene with a shRNA could inhibit FGF-2-induced cell migration. Pharmacological inhibition of CuSO_4_, also reduced FGF-2-induced cell migration in both MDA-MB-231 and Bcap-37 cells. Interestingly, compared to cells treated with the lentiviral shRNA, AQP3 expression was not notably affected when using CuSO_4_ (data not shown), indicating that the inhibitory effect of CuSO_4_ is transient, and that CuSO_4_ may not necessarily enter cells to exert its suppressing effect on AQP3 expression.

Our findings that AQP3 facilitates FGF-2-induced cell migration in breast cancer cells are consistent with the recent reports that AQPs is involved in many physiological and pathological functions [5]–[9], including cell migration. These results suggest that AQP3, another AQP besides AQP1 and AQP5, contributes to FGF-2-induced cell migration in human breast cancer cells.

The results showed that FGF-2 up-regulates AQP3 expression and enhances cell migration in MDA-MB-231 and Bcap-37 breast cancer cells. However, the mechanisms through which FGF-2 induces AQP3 expression are still under investigated. In the present study, we found that FGFR kinase inhibitor PD173074 significantly inhibited AQP3 expression, as well as cell migration in both MDA-MB-231 and Bcap-37 breast cancer cells, adding support to the putative role of FGFR in cell migration and AQP3 expression in human breast cancers.

The inhibitory effects of PI3K inhibitor LY294002, MEK1/2 inhibitor PD98059, and transient induction of AKT and ERK1/2 phosphorylation by FGF-2 suggested important roles of PI3K and ERK signaling pathways in FGF-2-induced AQP3 expression and cell migration. In addition, the finding that MEK1/2 inhibitor PD98059 exhibited more dramatic suppressing effects on AQP3 expression in comparison to PI3K inhibitor LY294002, highlighted the importance of ERK signal pathway. We speculate that the activation of FGFR initiates the downstream PI3K and ERK pathways and ultimately promotes FGF-2-induced cell migration through increased AQP3 expression.

In summary, we conclude that AQP3 is responsible for FGF-2-induced cell migration in human breast cancer cells, and that FGF-2 up-regulates AQP3 expression via the FGFR-PI3K and FGFR-ERK signal transduction pathways. These findings suggest a novel role of AQP3 in migration and metastasis of human breast cancer cells.
